# Genome-Wide Identification of PGRP Gene Family and Its Role in *Dendrolimus kikuchii* Immune Response Against *Bacillus thuringiensis* Infection

**DOI:** 10.3390/biology14121783

**Published:** 2025-12-13

**Authors:** Yanjiao Tang, Zizhu Wang, Qiang Guo, Xue Fu, Ning Zhao, Bin Yang, Jielong Zhou

**Affiliations:** 1 College of Biological Science and Food Engineering/Forest Resources Exploitation and Utilization Engineering Research Center for Grand Health of Yunnan Provincial Universities, Southwest Forestry University, Kunming 650224, China; tangyanjiao_bio@swfu.edu.cn (Y.T.); wzz2133992353@163.com (Z.W.); guoqiang@swfu.edu.cn (Q.G.); fxfuxue12345@163.com (X.F.); lijiangzhn@swfu.edu.cn (N.Z.); 2 Key Laboratory of Forest Disaster Warning and Control of Yunnan Province, Southwest Forestry University, Kunming 650224, China; 3 School of Biological and Chemical Science, Pu’er University, Pu’er 665000, China

**Keywords:** *Dendrolimus kikuchii*, PGRP, *Bacillus thuringiensis*, innate immunity, genome-wide identification, AMP

## Abstract

Peptidoglycan recognition proteins (PGRPs) are conserved pattern recognition receptors (PRRs) that play key roles in insect innate immunity by binding bacterial peptidoglycan (PGN) and activating downstream signaling pathways. The *Dendrolimus kikuchii*, a major defoliator of coniferous forests in southern China, has incompletely characterized immune defenses. This study systematically identified the PGRP gene family in *D. kikuchii* based on genome-wide data, identifying 10 PGRP genes with typical PGRP/Amidase_2 conserved domains, including 6 PGRP-S proteins and 4 PGRP-L proteins. Additionally, to further investigate the evolutionary relationships of these PGRP genes, a maximum likelihood (ML) phylogenetic tree was constructed using PGRP amino acid sequences from 6 different insect species, along with the 10 PGRP amino acid sequences from *D. kikuchii*. Phylogenetic analysis revealed that the DkikPGRP genes of *D. kikuchii* are distributed across distinct evolutionary branches and share high homology with PGRP genes from other insects, suggesting a close evolutionary relationship between the PGRP genes of *D. kikuchii* and those of other insect species. Transcriptome profiling revealed that *DkikPGRP-S1*, *-S2*, *-S3*, *-S4*, and *-S5* were upregulated in the midgut, fat body, and hemolymph after Bt infection, showing tissue- and time-specific immune responses. Functional assays using siRNA knockdown demonstrated distinct roles of *DkikPGRP-S4* and *DkikPGRP-S5*: *DkikPGRP-S5* mainly promoted antimicrobial peptide (AMP) expression, including attacin, lebocin, lysozyme, and cecropin, whereas *DkikPGRP-S4* showed a complex regulatory pattern, enhancing lebocin and lysozyme but suppressing attacin without affecting gloverin or cecropin. Silencing either gene significantly increased larval mortality upon Bt challenge. These results highlight the specialized immune regulatory functions of PGRPs in *D. kikuchii*, provide new insights into host–pathogen interactions, and suggest potential molecular targets for sustainable pest management strategies.

## Simple Summary

*Dendrolimus kikuchii* is a forest defoliator widely distributed in southern China, yet its mechanisms of defense against microbial challenge remain insufficiently characterized. We focused on peptidoglycan recognition proteins (PGRPs), molecules that help insects sense bacteria and start immune responses. Using the *D. kikuchii* genome, we identified 10 PGRP genes and examined how they respond to infection by *Bacillus thuringiensis* (Bt), a common biocontrol agent. Several short-form PGRPs were strongly activated in the midgut, fat body, and hemolymph after Bt challenge. When we silenced the expression of the key genes *DkikPGRP-S4* and *DkikPGRP-S5*, there was a significant increase in larval mortality under Bt infection. The two genes have different roles in regulating immunity in *D. kikuchii*. *DkikPGRP-S4* has both positive and negative regulatory effects on antimicrobial peptides, while *DkikPGRP-S5* primarily has a positive regulatory effect on antimicrobial peptides.

## 1. Introduction

PGRPs are key PRRs in insect innate immunity that specifically bind bacterial cell wall PGN and initiate downstream signaling [1]. First identified in the hemolymph and cuticle of the silkworm *Bombyx mori* in 1996 [2], PGRPs exhibit high binding affinity to PGN and trigger immune responses via the Toll/IMD pathways and the prophenoloxidase (PPO) cascade [1,3,4]. PGRPs are categorized as non-catalytic (recognition type) and catalytic types depending on the presence of amidase activity [5,6]. The former lack zinc-binding residues and primarily mediate pathogen recognition and signal activation; the latter retain Zn^2+^-binding residues (His42, His152, Cys160), as seen in species such as *Drosophila melanogaster*, where this Zn^2+^-binding structure is conserved in all catalytic PGRP [7]. This reveals the mechanism of PGN hydrolysis by catalytic PGRPs, with Zn^2+^ acting as a catalyst to facilitate the cleavage of the amide bond. Additionally, the zinc ion ligand, Cys160, has been shown to be essential for PGN hydrolysis [4]. These residues display amidase activity that can directly cleave PGN, function as bactericidal effectors, and negatively regulate immune signaling to prevent overactivation [4,5]. Insects generally possess short-type PGRPs (PGRP-S; typically secreted) and long-type PGRPs (PGRP-L; often membrane-associated) [8,9], with considerable interspecific variation in family size and functional diversification [10]. Previous studies have characterized the PGRP gene families in Lepidoptera species such as *Spodoptera frugiperda*, *Helicoverpa armigera*, and *Spodoptera exigua*. In *S. frugiperda*, PGRPs recognize both Lys-type and Dap-type PGNs, activating the Toll and IMD pathways to induce AMP expression [11,12]. Similarly, the PGRP family in *H. armigera* has undergone significant expansion, and differential expression in response to various pathogen infections suggests functional diversification, highlighting the adaptive versatility of its immune function [13]. In *S. exigua*, PGRP-LB plays a crucial role in antiviral immunity, further emphasizing the importance of PGRPs in the immune defense of Lepidoptera species [10]. These studies provide a valuable framework for understanding the immune function of PGRPs in *D. kikuchii*.

Bt is a widely used Gram-positive biocontrol agent that produces insecticidal parasporal crystals during sporulation [14,15]. After ingestion, Cry protoxins are activated in the larval midgut and form pores, disrupting the epithelial barrier and the gut microbiota, which can trigger host PGN recognition and immune cascades [16]. For Gram-positive Bt, PGRP-SA/SD cooperate with GNBP1 to recognize Lys-type PGN and activate the Toll pathway, inducing AMP expression [17,18], while the PPO cascade elicits melanization to restrict pathogen spread [2,19]; Dap-type PGN can activate the IMD pathway [20]. Certain catalytic PGRPs can directly hydrolyze PGN via amidase activity, thereby exhibiting bactericidal effects while modulating immune intensity [21].

*D. kikuchii* is among the most destructive Lepidoptera defoliators in coniferous forests of southern China and is listed as a major forestry quarantine pest [22]. It has a broad host range, including *Pinus yunnanensis*, *Casuarina equisetifolia*, *Pinus taiwanensis*, and *Pinus armandii*, and is distributed across Yunnan, Guizhou, Sichuan, Guangxi, and Fujian, with the most severe damage in Yunnan [23]. Larvae pass through multiple instars, with the 4th–5th instars showing the most intense feeding activity; a single larva can consume approximately 7486.6 cm of pine needles during its development (measured as cumulative needle length) [24]. High larval densities can rapidly defoliate extensive forest areas, producing so-called “smokeless fires” and thereby threatening ecosystem stability and regional biodiversity. In addition, the larvae bear urticating setae that may induce dermatitis and allergic reactions in humans and animals, raising concerns for public health. Recent genomic studies of *D. kikuchii* have revealed substantial expansions in immune-related genes, particularly within the Toll and IMD pathways [23], suggesting adaptation to complex environmental pressures and diverse pathogen challenges. Although significant progress has been made in the study of PGRPs in various Lepidoptera species, systematic research on the PGRP family in *D. kikuchii* remains relatively scarce. Here, we perform genome-wide identification and expression analyses of PGRPs in *D. kikuchii* to elucidate their roles in innate immune recognition and to inform the development of novel biocontrol strategies.

## 2. Materials and Methods

### 2.1. Insect Material, Bt Treatment, and Tissue Sampling

Pupae of *D. kikuchii* were collected in June 2020 from *P. yunnanensis* stands in Anning County (24°31′–25°6′ N, 102°8′–102°37′ E), Kunming City, Yunnan Province, China. The pupae were kept in insectary cages, where adults were allowed to mate and oviposit. Larvae were reared at 27.5 ± 2 °C, 75 ± 3% relative humidity (RH), and a photoperiod of 16 h light/8 h dark, and were fed with *P. yunnanensis* needles. Fifth-instar larvae of consistent size and in healthy condition were selected for experiments.

Bt (strain ACCC 10062) was obtained from the Agricultural Culture Collection of China (ACCC, Beijing, China). Based on preliminary assays, the concentration range causing 10–90% mortality was established. The Bt suspension was serially diluted 10-fold in phosphate-buffered saline (PBS) to prepare five concentrations, which were used to determine the 24 h median lethal concentration (LC_50_ = 5.7 × 10^5^ spores/mL) in fifth-instar larvae. Subsequently, larvae were treated with the LC_50_ suspension, with 120 larvae per treatment group and three biological replicates, while a 0.01 M PBS treatment served as the control. Tissue samples were collected at 6, 12, and 24 h after Bt treatment.

Hemolymph was collected by cutting the third abdominal legs, and hemocytes were centrifuged and quickly frozen in liquid nitrogen. The midgut and fat body tissues were dissected and immediately placed into RNA stabilization solution. These samples were stored at 4 °C for 24 h before transferring to −80 °C for long-term storage.

### 2.2. RNA Extraction, High-Throughput Sequencing, and Data Analysis

Total RNA was extracted from tissues using the Trizol method, and RNA concentration was measured using the Nanodrop2000 (Thermo Fisher Scientific, Waltham, MA, USA). RNA integrity was assessed by agarose gel electrophoresis and the Agilent2100 system (Agilent Technologies, Santa Clara, CA, USA), with the RNA Integrity Number (RIN) calculated.

Library construction and sequencing were performed by Shanghai Major Bio-Pharmaceutical Technology Co., Ltd. (Shanghai, China), using the Illumina HiSeq 6000 platform for high-throughput sequencing. Raw sequencing data were processed using Fastp (v0.23.4) for quality control, including adapter trimming and removal of low-quality bases [25]. The cleaned data were aligned to the *D. kikuchii* reference genome (uploaded by our research group to NCBI GenBank: GCA_019925095.2) using HiSat2 (v2.2.1) [26]. Gene expression levels were quantified using RSEM software (v1.3.3), with results normalized to TPM (Transcripts per Million) to ensure consistent total expression across samples. Differential expression analysis was performed using DESeq2 (v1.32.0), with the thresholds for differentially expressed genes set at |log2FC| ≥ 1 and adjusted *p*-value < 0.05 [27].

### 2.3. Identification of the PGRP Gene Family

The *D. kikuchii* genome data used in this study was sequenced by our research group and uploaded to NCBI (GenBank: GCA_019925095.2). Using the TBtools program (v2.330), CDSs, and protein sequences were extracted from the whole-genome sequence of *D. kikuchii*. PGRP sequences from six species, including *D. melanogaster*, *Bombyx mori*, *Manduca sexta*, *Apis mellifera*, *Anopheles gambiae*, and *Trichoplusia ni* were retrieved from NCBI [28]. Using the PGRP sequences of the six species retrieved as the query sequences, the TBtools (v2.330) program was used to identify the homologous *D. kikuchii* PGRP sequences. Only sequences with an e-value ≤ 1.0 × 10^−5^ and a minimum length of 100 amino acids were selected. Candidate sequences were validated by NCBI BLASTp (https://blast.ncbi.nlm.nih.gov/Blast.cgi (accessed on 20 July 2025) default settings. To identify all potential sequences of DkikPGRP genes, we downloaded the HMM profile (PF01510) of the domain from the Pfam database with an e-value < 0.00 and other uses the default parameters. The sources of insect data used for PGRP comparisons are provided in Table S1.

### 2.4. Characterization of PGRP Genes

The PGRP domains of candidate sequences were predicted using the Pfam database (https://www.ebi.ac.uk/interpro/pfam/ (accessed on 26 July 2025), and conserved domains were further verified through NCBI-CDD (https://www.ncbi.nlm.nih.gov/cdd/ (accessed on 26 July 2025) and InterPro (http://www.ebi.ac.uk/interpro/ (accessed on 26 July 2025). The physicochemical properties of proteins were predicted using ExPASy (http://web.expasy.org/protparam/ (accessed on 27 July 2025) [29], including molecular weight (MW), isoelectric point (pI), grand average of hydropathicity (GRAVY), instability index, and aliphatic index. Conserved amino acid residues in PGRP sequences were visualized using WebLogo3 (http://weblogo.threeplusone.com/ (accessed on 27 July 2025). Signal peptides at the N-terminus were predicted with SignaIP 6.0 (https://services.healthtech.dtu.dk/services/SignalP-6.0/ (accessed on 27 July 2025), and transmembrane regions were predicted using TMHMM (http://www.cbs.dtu.dk/services/TMHMM/ (accessed on 27 July 2025). The protein sequences of DkikPGRP genes were submitted to MEME (http://meme-suite.org/meme/ (accessed on 27 July 2025) to identify conserved motifs. Motif and domain distribution maps were generated with TBtools (v2.330). In addition, TBtools was used to visualize the chromosomal locations of DkikPGRP genes.

### 2.5. Multiple Sequence Alignment and Phylogenetic Analysis

Multiple sequence alignment of PGRP sequences was performed using MAFFT (https://mafft.cbrc.jp/alignment/server/index.html (accessed on 28 July 2025). PGRP sequences of *D. melanogaster*, *B. mori*, *S. frugiperda*, *T. castaneum*, *A. mellifera*, and *D. plexippus* were downloaded from the NCBI database (http://www.ncbi.nlm.nih.gov/ (accessed on 28 July 2025) to identify the PGRP gene families of these species (Table S2). For the phylogenetic analysis, the *D. kikuchii* PGRP sequences were aligned using the Muscle tool integrated in MEGA 11. After alignment, a maximum likelihood (ML) tree was constructed using the default parameters, and 1000 bootstrap replicates were performed to assess the robustness of the tree. Finally, the tree was beautified using the iTOL online tool (accessed on 28 July 2025).

### 2.6. siRNA Interference and Functional Validation

To investigate the immune functions of *PGRP-S4* and *PGRP-S5* in *D. kikuchii*, specific siRNAs were synthesized according to their gene sequences by Sangon Biotech (Shanghai) Co., Ltd. (Shanghai, China) sing the phosphoramidite solid-phase synthesis method. A random nucleotide sequence was used as a negative control (siNC). Fifth-instar larvae were randomly divided into three groups and injected with siPGRP-S4, siPGRP-S5, or siNC, with 90 larvae per group and three biological replicates. The primer sequences for the siRNA experiment are provided in Table S3.

The siRNA solution was prepared at 2 µg/µL (one OD of siRNA dissolved in 20 µL of DEPC-treated water). Using a sterile microsyringe, 2 µL of the solution was injected into the hemocoel of larvae anesthetized on ice. At 24 h post-injection, 30 larvae from each group were collected for RNA extraction to evaluate interference efficiency. The β-actin gene was used as the housekeeping gene in this study for normalization. Its corresponding gene ID in the *D. kikuchii* genome is *Dkikuchii*_LG18_G00009, which was retrieved from the genome annotation file. This gene was selected due to its stable expression across different tissues and conditions. β-actin was employed as an internal control for qRT-PCR to normalize the expression of target genes, ensuring reliable comparison of gene expression levels. The remaining larvae were re-anesthetized on ice and injected with a Bt suspension (2 µL per larva, 5.7 × 10^5^ spores/mL). At 12 h post-infection, the transcript levels of downstream AMP genes (cecropin, gloverin, lysozyme, lebocin, and attacin) were quantified by qRT-PCR. At 24 h, larval mortality in each group was recorded to evaluate the effects of siRNA-mediated knockdown of the two PGRP genes on the immune response of *D. kikuchii* and on the virulence of Bt. The qRT-PCR primer information is provided in Table S4.

## 3. Results

### 3.1. Genome-Wide Identification of PGRP Genes in Dendrolimus kikuchii

The PGRP gene family was systematically identified in seven species, including *D. melanogaster*, *B. mori*, *S. frugiperda*, *T. castaneum*, *A. mellifera*, *D. plexippus*, and *D. kikuchii*. The results showed clear differences in the number of PGRP genes among these species: 13 in *D. melanogaster*, 12 in *B. mori*, 12 in *S. frugiperda*, 9 in *T. castaneum*, 10 in *D. kikuchii*, 4 in *A. mellifera*, and 5 in *D. plexippus* [9,12,30].

In the *D. kikuchii* genome, a total of 10 PGRP genes were identified, including six short-type (*DkikPGRP-S1*, *DkikPGRP-S2*, *DkikPGRP-S3*, *DkikPGRP-S4*, *DkikPGRP-S5*, and *DkikPGRP-S6*) and four long-type (*DkikPGRP-L1*, *DkikPGRP-L2*, *DkikPGRP-LC*, and *DkikPGRP-LE*) members (Table 1). Homology analysis revealed that the PGRP genes of *D. kikuchii* shared relatively high sequence similarity (39.4–78.5%) with those of other insects such as *Trichoplusia ni*, *Galleria mellonella*, and *Samia ricini*, indicating strong conservation of sequence and structure across insect PGRPs (Table 1).

### 3.2. Characterization of Dendrolimus kikuchii PGRPs

Based on the complete amino acid sequences of the 10 identified *D. kikuchii* PGRPs, their CDS lengths and physicochemical properties were systematically analyzed (Table S5). The results showed that the protein lengths ranged from 186 to 463 amino acids, with predicted molecular weights of 20.32–53.34 kDa, and theoretical isoelectric points (pI) of 5.48–8.85. The instability index values ranged from 26.68 to 52.66, among which *DkikPGRP-L1*, *DkikPGRP-S2*, *DkikPGRP-S3*, *DkikPGRP-LC*, and *DkikPGRP-LE* were below 40 and therefore classified as stable proteins, while the remaining members were predicted to be unstable. indicating relatively high thermostability across the PGRP family. The GRAVY index values ranged from –0.285 to 0.075, with eight PGRPs (*DkikPGRP-L1*, *-L2*, *-LC*, *-LE*, *-S1*, *-S3*, *-S4*, and *-S6*) identified as hydrophilic, whereas *DkikPGRP-S2* and -*S5* were predicted to be hydrophobic.

### 3.3. Motif Patterns, Conserved Domains, and Chromosomal Localization of PGRPs

**Figure 1:**
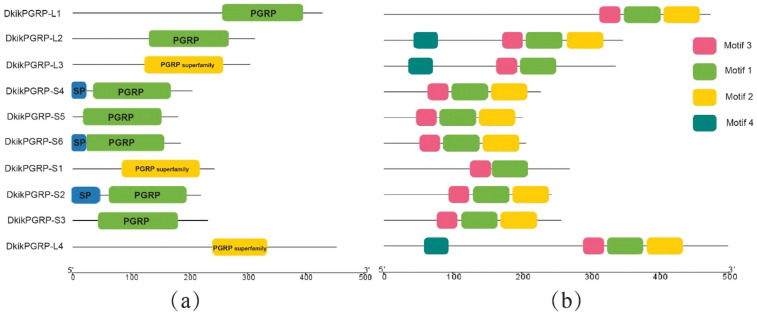
Domain (**a**) and motif (**b**) structure diagrams of PGRPs in *D. kikuchii*. (**a**) Conserved domains of ten PGRPs. (**b**) Motif organization. SP, signal peptide; PGRP, peptidoglycan recognition protein domain; PGRP superfamily, peptidoglycan recognition protein superfamily.

Analysis using the CDD database revealed that all ten *D. kikuchii* PGRPs contained the typical PGRP domain. Among them, *DkikPGRP-LC*, *DkikPGRP-S1*, and *DkikPGRP-LE* were assigned to the PGRP superfamily domain (Figure 1a). Transmembrane domain prediction further showed that *DkikPGRP-L1*, *-L2*, *-LC*, and *-LE* possessed three, one, one, and one transmembrane domains, respectively, implying that these proteins may be transported extracellularly via alternative mechanisms or exist as membrane-associated proteins. Based on the SignaIP 6.0 predictions, the N termini of *DkikPGRP-S2*, *-S4* and *-S6* display signal peptide features, which is consistent with their annotation as secreted PGRPs, whereas no signal peptide was detected in the currently available amino acid sequences of *DkikPGRP-S1*, *-S3* and *-S5*. Given that short PGRPs in other insects usually possess an N-terminal signal peptide, these predictions should be interpreted with caution, and the 5′ regions of these genes remain to be validated experimentally. Taken together, the signal peptide and transmembrane helix predictions suggest that *D. kikuchii* PGRPs may comprise secreted, membrane-bound and non-membrane forms, although the precise subcellular localization of several members still requires further investigation.

Motif analysis showed that all *D. kikuchii* PGRPs contained Motifs 1–3, whereas *DkikPGRP-L2* and *-LE* contained all four identified motifs (Figure 1b). These findings indicate that, although the domains and subcellular localizations of different PGRPs varied, their core motifs remain highly conserved.

**Figure 2:**
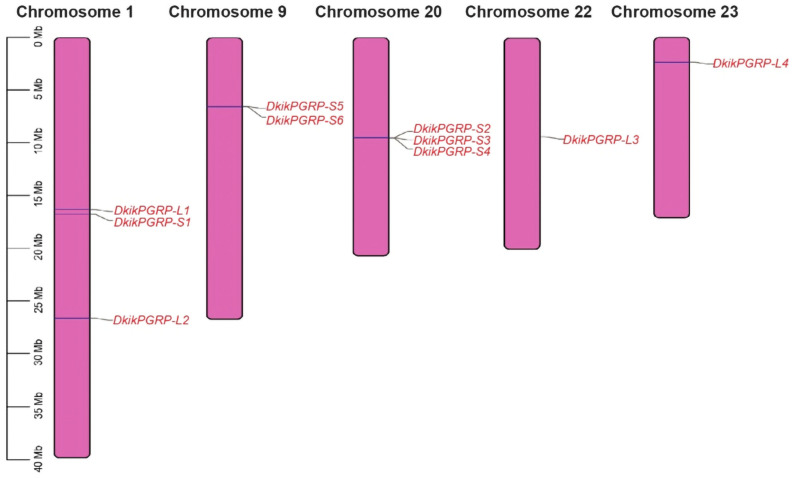
Chromosomal distribution of DkikPGRP genes in the *D. kikuchii* genome. The figure shows the chromosomal distribution of ten DkikPGRP genes in *D. kikuchii*. Numbers indicate chromosome IDs, and the left scale represents chromosome length (Mb).

The chromosomal localization of the DkikPGRPs is shown in Figure 2. A total of ten genes were mapped to five chromosomes, with chromosomes 1 and 20 each harboring the highest number of genes (three genes each). Chromosome 9 contained two genes, while chromosomes 22 and 23 each contained one gene.

### 3.4. Domain Analysis of PGRPs

**Figure 3:**
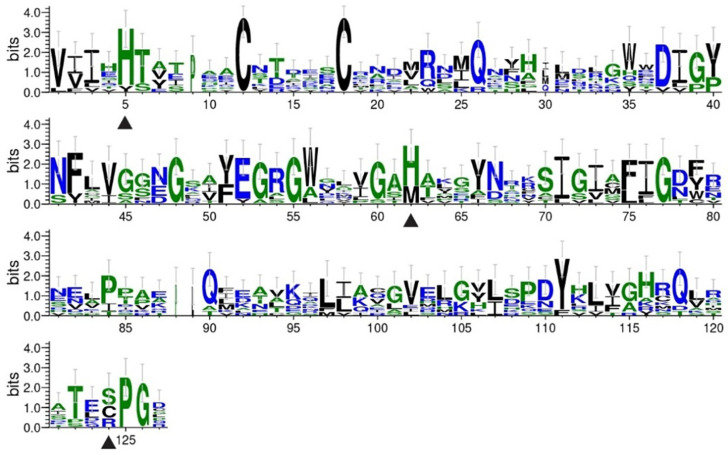
Amino Acid Conservation of PGRP Domains in *D. kikuchii*. Triangles Indicate Zn^2+^-Binding Sites.

Amino acid conservation within the functional domains of *D. kikuchii* PGRPs was analyzed using WebLogo (v3.7.11), revealing strong conservation at several key functional sites (Figure 3). These conserved residues constitute the structural basis for immune recognition and catalytic activity of PGRPs. Specifically, Zn^2+^-binding sites were identified at His5, His62, and Cys124; amidase catalytic residues included His5, Tyr40, His62, Thr122, and Cys124; and additional substrate-binding sites were also detected. These results indicate that *D. kikuchii* PGRPs exhibit evolutionary conservation at critical residues, underscoring their functional significance in immune recognition and catalysis.

The multiple sequence alignment of PGRP domain sequences (Figure S1) showed that *DkikPGRP-S2*, *DkikPGRP-S3*, and *DkikPGRP-S4* fully retained the five key catalytic residues required for amidase activity (His5, Tyr40, His62, Thr122, and Cys124), suggesting that they possess Amidase_2 activity and can hydrolyze the amide bond between N-acetylmuramic acid and L-alanine in bacterial peptidoglycan, thereby exerting direct bactericidal effects against Gram-positive bacteria. In particular, His5, His62, and Cys124 function as Zn^2+^-binding sites that are critical for catalysis. By contrast, mutations in certain residues may reduce catalytic activity; for example, in *DkikPGRP-S5*, *DkikPGRP-S6*, and *DkikPGRP-L2*, Cys124 is replaced by Ser124, which may impair Zn^2+^-binding and amidase function.

### 3.5. Phylogenetic Analysis of the PGRP Family

**Figure 4:**
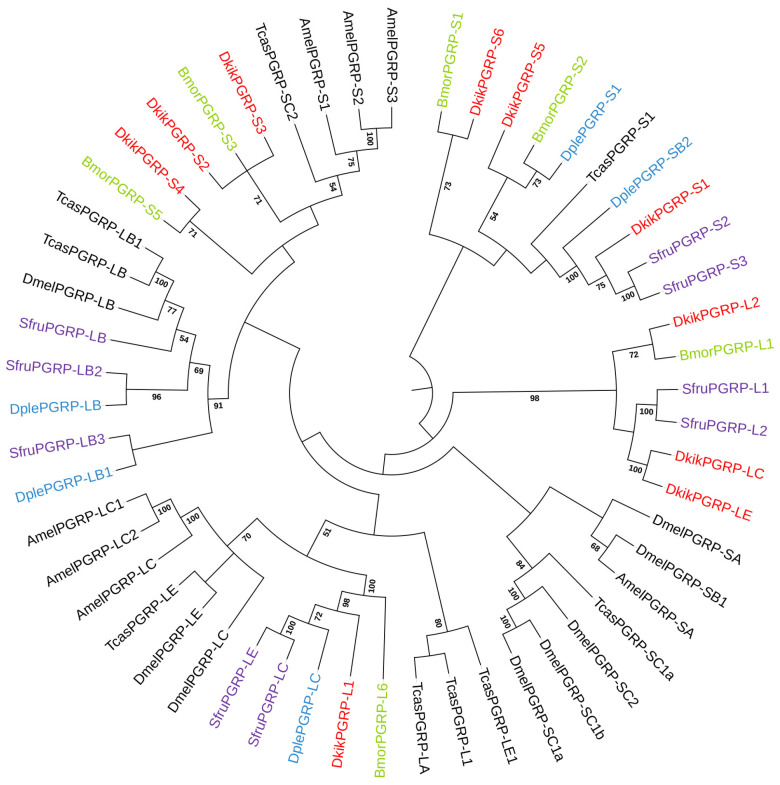
Phylogenetic analysis of the PGRP family in *D. kikuchii*. The maximum-likelihood tree was constructed in MEGA 11.0 using PGRP amino acid sequences from *D. kikuchii*, *A. mellifera*, *B. mori*, *D. melanogaster*, *D. plexippus*, *S. frugiperda*, and *T. castaneum*. The bootstrap test replicate number was set to 1000. Numbers at the internal nodes indicate bootstrap support values (%), and only values ≥ 50% are shown. Red font represents *D. kikuchii* PGRP genes.

To explore the evolutionary relationships of the PGRP family, a neighbor-joining phylogenetic tree was constructed using PGRP amino acid sequences from seven insect species (Figure 4). The ten DkikPGRP of *D. kikuchii* were distributed across distinct clades and clustered with homologs from other Lepidoptera insects. For instance, *DkikPGRP-S1* clustered with *SfruPGRP-S2* and *SfruPGRP-S3*; *DkikPGRP-S2* and *DkikPGRP-S3*, along with *BmorPGRP-S3*, cluster together in one branch, indicating a close evolutionary relationship between these genes. Similarly, *DkikPGRP-S4* and *BmorPGRP-S5* are grouped in another branch, suggesting they share a common evolutionary ancestor. *DkikPGRP-S5* shows a clear evolutionary relationship with other PGRP genes from *D. kikuchii* and closely related species. Its placement in the phylogenetic tree suggests it is genetically distinct but shares a common ancestor with other PGRP family members. This positions *DkikPGRP-S5* as an important, potentially unique gene in the immune response of *D. kikuchii*; *DkikPGRP-S6* was closely related to *BmorPGRP-S1*. These groupings highlight the conserved nature of certain PGRP genes across different species, reflecting similar roles in immune defense. Among the long-type proteins, *DkikPGRP-L1* clustered with *DplePGRP-LC*, *SfruPGRP-LC*, *SfruPGRP-LE*, and *BmorPGRP-L6*; *DkikPGRP-L2* with *BmorPGRP-L1*; *DkikPGRP-LC* and *DkikPGRP-LE* also clustered with *SfruPGRP-L1* and *SfruPGRP-L2*. Overall, *DkikPGRPs* exhibited strong homology with Lepidoptera PGRPs, highlighting their evolutionary conservation.

### 3.6. Tissue-Specific Transcriptional Expression of PGRP Genes Under Bt Infection

**Figure 5:**
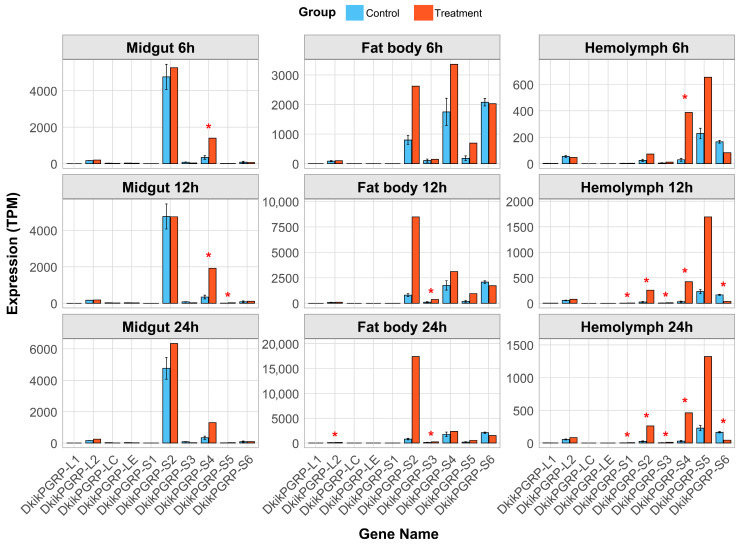
Expression of PGRP genes in different tissues of *D. kikuchii* under Bt infection. The expression levels of PGRP genes in the midgut, fat body, and hemolymph were measured at 6, 12, and 24 h post-Bt infection using qRT-PCR. The red and blue bars represent the treatment and control groups, respectively. Asterisks denote statistically significant differences: * *p* < 0.05.

Based on transcriptomic data, the expression patterns of PGRP genes in the midgut, fat body, and hemolymph of *D. kikuchii* were comparatively analyzed under Bt infection (Figure 5). Among the ten identified PGRP genes, the four long-type genes (*DkikPGRP-L1*, *-L2*, *-LC*, and *-LE*) showed no significant response to Bt infection across the tested tissues, with expression levels comparable to those of the control group. In contrast, the six short-type genes (*DkikPGRP-S1–S6*) exhibited pronounced changes across different tissues and time points, suggesting that they play more active roles in immune responses.

Among these, *DkikPGRP-S5* showed the most significant upregulation, with consistently elevated expression in the midgut, fat body, and hemolymph at nearly all examined time points. *DkikPGRP-S2* and *DkikPGRP-S3* were predominantly upregulated in the fat body and hemolymph, while *DkikPGRP-S4* was markedly induced in the midgut and hemolymph. By contrast, *DkikPGRP-S1* and *DkikPGRP-S6* exhibited significant responses only in the hemolymph. Further analysis revealed that as Bt infection progressed, the number of short-type PGRPs involved in immune responses increased, indicating a cooperative role in defense against Bt. Collectively, these results suggest that short-type PGRPs play a critical role in the immune defense of *D. kikuchii* against the Gram-positive bacterium Bt, with responses exhibiting clear tissue specificity and temporal dependence. These findings are consistent with recent reports that Bt infection can induce enhanced immune responses in Lepidoptera pests [31].

### 3.7. Functional Analysis of DkikPGRP-S4 and DkikPGRP-S5 in Immune Regulation

#### 3.7.1. Effects of siRNA on Gene Expression and Bt Virulence

Based on the transcriptional expression analysis of PGRP genes in different tissues under Bt infection, both *DkikPGRP-S4* and *DkikPGRP-S5* were significantly upregulated in multiple immune-related tissues, suggesting that they may play critical roles in immune responses. Domain analysis further indicated that *DkikPGRP-S4* possesses a complete set of amidase catalytic residues, whereas *DkikPGRP-S5* carries a mutation at Cys124, which is substituted by Ser124, potentially impairing its catalytic activity. To clarify their functional differences during Bt infection, siRNA-mediated RNAi experiments targeting *DkikPGRP-S4* and *DkikPGRP-S5* were performed to assess their roles in downstream immune responses and Bt virulence.

**Figure 6:**
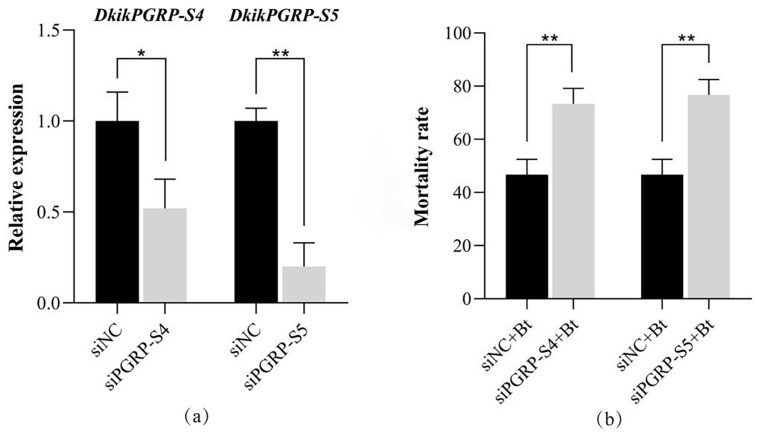
siRNA efficiency and effects of *DkikPGRP-S4* and *DkikPGRP-S5* on Bt virulence. (**a**) Efficiency of siRNA interference on *DkikPGRP-S4* and *DkikPGRP-S5* expression levels, measured by qRT-PCR at 24 h post-injection. (**b**) Mortality of larvae infected with Bt at 24 h after siRNA treatment. Data are presented as mean ± SD. Different asterisks indicate significant differences; * and ** indicate significance at *p* < 0.05 and *p* < 0.01, respectively.

According to previous studies, the target gene silencing can be detected within 24 h after RNA interference (RNAi) injection in insects [32,33]. Therefore, the silencing efficiency was assessed at 24 h post-injection in this study. The results (Figure 6a) showed that 24 h after siRNA treatment, the relative expression levels of *DkikPGRP-S4* and *DkikPGRP-S5* were significantly downregulated. Compared to the control group si-NC, the silencing efficiencies of si*DkikPGRP-S4* and si*DkikPGRP-S5* were 48% and 80%, respectively. This indicates that the transcriptional level of *DkikPGRP-S5* was more significantly inhibited by siRNA (*p* < 0.01), with a greater effect than the silencing effect of siRNA on *DkikPGRP-S4* (*p* < 0.05).

After 24 h of siRNA interference in *D. kikuchii*, the larvae were injected with Bt bacterial solution, and the effect of siRNA-mediated target gene silencing on Bt toxicity was analyzed 24 h later. As shown in Figure 6b, effective inhibition of *DkikPGRP-S4* and *DkikPGRP-S5* mRNA levels by siRNA treatment led to a significant increase in the mortality rate of *D. kikuchii* larvae following Bt infection (*p* < 0.01). Although there was a considerable difference in the silencing efficiency of the two genes, both siRNAs exacerbated the toxicity of Bt on the larvae, with little difference in the lethal effect. These results indicate that both *DkikPGRP-S4* and *DkikPGRP-S5* play a role in regulating Bt toxicity.

#### 3.7.2. Regulation of Downstream AMP Gene Expression by *DkikPGRP-S4* and *DkikPGRP-S5*

**Figure 7:**
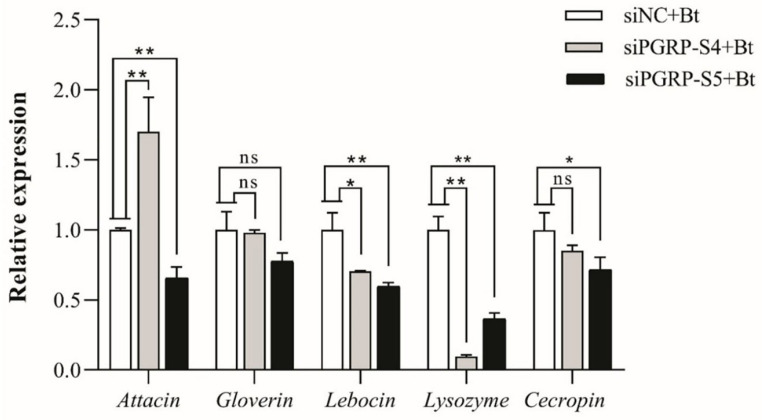
Regulation of AMP gene expression by *DkikPGRP-S4* and *DkikPGRP-S5* after siRNA interference. After siRNA treatment and Bt injection, AMP gene expression levels were measured at 12 h post-infection using qRT-PCR. Data are shown as mean ± SD. * and ** indicate significance at *p* < 0.05 and *p* < 0.01, respectively; ns indicates no significant difference. The expression levels of each gene were normalized to its respective siNC + Bt control group, which was set as 1.0.

After siRNA treatment for 24 h, larvae were injected with Bt, and the mRNA levels of downstream AMP genes (attacin, gloverin, lebocin, lysozyme, and cecropin) were measured 12 h post-infection (Figure 7).

The results showed that silencing *DkikPGRP-S4* significantly downregulated the expression of lebocin and lysozyme, while significantly upregulating attacin. This indicates that *DkikPGRP-S4* exerts both positive and negative regulatory effects on different AMP genes. No significant changes were observed in gloverin and cecropin, indicating that these two genes may not be regulated by *DkikPGRP-S4*. In contrast, silencing *DkikPGRP-S5* resulted in significant downregulation of all AMP genes (attacin, lebocin, lysozyme, and cecropin) except gloverin, suggesting that it primarily functions as a positive regulator of AMP expression, while gloverin may not be under its control. Collectively, these findings indicate that *DkikPGRP-S4* and *DkikPGRP-S5* contribute to the defense of *D. kikuchii* against Bt infection by regulating the expression of downstream AMP genes.

In summary, both *DkikPGRP-S4* and *DkikPGRP-S5* contribute to the immune defense of *D. kikuchii* against Bt by regulating the expression of downstream AMP genes. Specifically, *DkikPGRP-S4* likely exerts its function through amidase activity that directly weakens Bt, whereas *DkikPGRP-S5*, despite lacking catalytic activity, enhances the defense response by recognizing Bt and inducing AMP expression. The loss of their synergistic action ultimately compromises immune defense, significantly increases larval susceptibility to Bt, and consequently leads to a marked rise in larval mortality.

## 4. Discussion

PGRPs are a conserved family of PRRs that recognize bacterial cell wall PGN, thereby activating the insect innate immune system and playing key roles in defense against pathogen invasion [21,28]. Most studies to date have focused on model insects such as *D. melanogaster* and *B. mori*, whereas systematic investigations of PGRPs in non-model insects remains relatively limited [22,23].

In this study, ten PGRP genes were identified in the genome of *D. kikuchii*, including four long-type (L-type) and six short-type (S-type) members. The number of genes was comparable to that in *T. castaneum* (10) and *H. armigera* (9) [34], suggesting that the PGRP family in *D. kikuchii* is similar in scale to that of other insects, regardless of their taxonomic classification [35]. Phylogenetic analysis revealed that these genes exhibit close evolutionary relationships with PGRPs from other insects, and all contain the typical Amidase_2 conserved domain, confirming that they are canonical members of the PGRP family. Furthermore, *DkikPGRP-S2*, *DkikPGRP-S3*, and *DkikPGRP-S4* clustered together phylogenetically and were tandemly distributed on chromosome 20, whereas *DkikPGRP-S5* and *DkikPGRP-S6* were located adjacently on chromosome 9, suggesting tandem duplication events at the chromosomal level. Such a phenomenon of “tandem distribution with functional convergence” has also been reported in other insects [34], indicating that gene expansion plays an important role in shaping the diversity of immune-related genes.

PGRPs can participate in immune responses through different mechanisms: catalytic PGRPs retain Zn^2+^-binding sites and exhibit amidase activity, enabling them to directly hydrolyze PGN and negatively regulate immune responses [6,36,37]; whereas non-catalytic PGRPs, which have lost the Zn^2+^-binding residues, can only bind PGN and activate immune signaling pathways [3,38]. For example, *Drosophila PGRP-SB1* can rapidly kill Gram-positive bacteria in the presence of Zn^2+^ [38], whereas *B. mori PGRP-S4* has been shown to directly hydrolyze *Escherichia coli* PGN and disrupt its cell surface [39]. Consistently, structural predictions in this study indicated that the DkikPGRP family contains several functional amino acid residues, with His5, His62, and Cys124 identified as critical Zn^2+^-binding sites. *DkikPGRP-S2*, *DkikPGRP-S3*, and *DkikPGRP-S4* were found to possess complete catalytic sites, suggesting typical amidase activity. In contrast, *DkikPGRP-S5* and *DkikPGRP-S6* carry a Cys124-to-Ser124 substitution, which may impair their ability to hydrolyze PGN, resembling previously reported loss-of-function mutations [3].

Transcriptomic data and RNAi functional validation further demonstrated that *DkikPGRP-S4* and *DkikPGRP-S5* play critical roles in the immune defense of *D. kikuchii*. Silencing of *DkikPGRP-S4* resulted in downregulation of Lebocin and Lysozyme expression, whereas attacin was upregulated, indicating a bidirectional regulatory role in AMPs. In contrast, silencing of *DkikPGRP-S5* led to significant downregulation of most AMPs (attacin, lebocin, lysozyme, and cecropin), except for gloverin, suggesting that it primarily functions as a positive regulator of AMP expression. This finding is consistent with previous studies showing that *BmorPGRP-S5* in the *B. mori* AMP synthesis [40], and also aligns with results from *Plutella xylostella* exposed to Cry1Ac toxin, in which PGRPs were implicated in host immune regulation [41]. Moreover, silencing of either gene significantly increased larval mortality following Bt infection, further confirming their essential roles in antibacterial defense.

Notably, this study revealed that *DkikPGRP-S4* is more likely to function through its amidase activity by directly hydrolyzing PGN, whereas *DkikPGRP-S5*, despite lacking amidase activity, can recognize PGN and activate the Toll and IMD pathways to induce downstream AMP expression (e.g., Cecropin), thereby enhancing immune defense. This hypothesis is consistent with RNAi experiments conducted in *Antheraea pernyi* and *P. xylostella*, where silencing of PGRPs resulted in significant downregulation of AMP expression and increased host susceptibility to pathogens [41,42].

The most novel finding in our study is the complex regulatory pattern of *DkikPGRP-S4*, which both activates and represses the expression of different AMPs. This dual role contrasts with the canonical model of PGRPs in *D. melanogaster*, where catalytic PGRPs are generally considered negative regulators of immune responses [43]. In *Drosophila*, catalytic PGRPs activate the immune pathway by recognizing PGN, but subsequently limit immune activation to prevent overreaction, typically by repressing certain immune responses [5].

However, our findings suggest that *DkikPGRP-S4* in *D. kikuchii* exhibits a more complex role, modulating AMP expression both positively and negatively. Specifically, *DkikPGRP-S4* activates the expression of attacin, while repressing the expression of lebocin and lysozyme, indicating that its function is more intricate than simply acting as a repressor. This suggests that *D. kikuchii* may have evolved a more complex immune mechanism. The complex regulatory pattern of *DkikPGRP-S4* may reflect a more adaptive strategy, balancing immune activation and repression to enable *D. kikuchii* to more effectively respond to various pathogens, such as Bt. The broader implication of this finding is that catalytic PGRPs may have evolved different roles across insect species, providing a more flexible and refined immune response than previously thought.

It should be noted that, although SignaIP 6.0 did not predict N-terminal signal peptides in the currently available sequences of *DkikPGRP-S1*, *-S3*, and *-S5*, short PGRPs in other insects usually possess such signal peptides. In the present study, we did not directly verify the 5′-end completeness or subcellular localization of these PGRPs by 5′RACE or localization assays, which represents a limitation of our work. Future studies should therefore perform 5′RACE and protein localization analyses to obtain full-length sequences and clarify their precise subcellular distribution and functions within cells.

Therefore, this study not only elucidates the conservation and diversity of the PGRP gene family in *D. kikuchii* but also clarifies their critical molecular roles in resisting Bt infection. These findings advance our understanding of the structure and function of insect PGRPs and highlight potential molecular targets for the sustainable management of forestry pests such as *D. kikuchii*. With the increasing application of RNA interference (RNAi) technology in Lepidoptera pests [44], this approach shows great promise for immune regulation research and pest control in *D. kikuchii*, offering new strategies for future integrated pest management.

## 5. Conclusions

This study systematically identified 10 PGRP family members in *D. kikuchii* (6 short-type PGRPs and 4 long-type PGRPs), all containing typical PGRP/Amidase_2 domains. The genes are highly conserved at the sequence level, with several members showing evidence of tandem expansion. Transcriptome analysis revealed that five short PGRP genes (*DkikPGRP-S1/S2/S3/S4/S5*) play important roles in the innate immune response to Bt infection, exhibiting tissue specificity and temporal dependence. siRNA-mediated knockdown experiments indicated functional differences between *DkikPGRP-S4*, which retains full amidase activity, and *DkikPGRP-S5*, which has mutations at the active site. However, silencing either gene significantly increased larval mortality following Bt infection, suggesting that these PGRP genes are promising molecular targets for controlling *D. kikuchii* damage.

## Figures and Tables

**Table 1:** Identification of PGRP genes in *D. kikuchii* and their homology with other insects.

Gene ID	PGRP Gene Name	Best-Matched Insect Gene (Species/Accession Number)	Amino Acid Identity (%)
D.kikuchii_LG01_G00316	*DkikPGRP-L1*	*Trichoplusia ni* (XP-026730977.1)	55.8
D.kikuchii_LG01_G00520	*DkikPGRP-L2*	*Trichoplusia ni* (XP-026738966.1)	56.0
D.kikuchii_LG22_G00198	*DkikPGRP-LC*	*Trichoplusia ni* (XP-026739039.1)	42.5
D.kikuchii_LG23_G00044	*DkikPGRP-LE*	*Trichoplusia ni* (XP-026739039.1)	39.4
D.kikuchii_LG01_G00331	*DkikPGRP-S1*	*Galleria mellonella* (XP-026757906.1)	54.8
D.kikuchii_LG20_G00235	*DkikPGRP-S2*	*Samia ricini* (BAF03520.1)	65.5
D.kikuchii_LG20_G00237	*DkikPGRP-S3*	*Helicoverpa armigera* (AHL58837.1)	78.5
D.kikuchii_LG20_G00236	*DkikPGRP-S4*	*Leguminivora glycinivorella* (AXS59129.1)	64.6
D.kikuchii_LG09_G00102	*DkikPGRP-S5*	*Hyposmocoma kahamanoa* (XP-026322322.1)	57.7
D.kikuchii_LG09_G00101	*DkikPGRP-S6*	*Trichoplusia ni* (XP-026737257.1)	64.1

## Data Availability

The data presented in the study are deposited in the NCBI repository; the genome sequence and annotation accession number is JAHHIN010000000; the transcriptome analysis accession numbers are SRR15334172-SRR15334183 and SRR15927891-SRR15927903. The datasets generated and/or analyzed during the current study are available from the corresponding author upon reasonable request.

## Supplementary Materials

The following supporting information can be downloaded at: https://www.mdpi.com/article/10.3390/biology14121783/s1, Table S1: Insect Genome Data for PGRP Identity (NCBI); Table S2: List of PGRP Genes and Corresponding GenBank Accession Numbers Used in the Phylogenetic Analysis; Table S3: siRNA Primer Sequence Information; Table S4: Fluorescence PCR Primer Information; Table S5: Physicochemical properties of PGRPs in *Dendrolimus kikuchii*; Figure S1. Multiple sequence alignment of PGRP domains in *Dendrolimus kikuchii*.

## Abbreviations

The following abbreviations are used in this manuscript:

PGRP	Peptidoglycan recognition proteins
PRRS	Pattern Recognition Receptors
PGN	Peptidoglycan
PPO	Prophenoloxidase
Bt	*Bacillus thuringiensis*
PBS	Phosphate-Buffered Saline
AMP	Antimicrobial Peptide
siNC	siRNA Negative Control
CDS	Coding Sequence
CDD	Conserved Domain Database
mRNA	Messenger RNA
siRNA	Small Interfering RNA
qRT-PCR	Real-Time Quantitative Reverse Transcription PCR
5′-RACE	Rapid Amplification of cDNA Ends
