# Supplementary material for: Genome-Wide Identification of PGRP Gene Family and Its Role in Dendrolimus kikuchii Immune Response Against Bacillus thuringiensis Infection
Source: Biology (Basel). 2025 Dec 13;14(12):1783. doi: 10.3390/biology14121783 (PMC12730284; doi:10.3390/biology14121783)
Supplement: Supplementary file 1 [file biology-14-01783-s001.zip › Figure S1. Multiple sequence alignment of PGRP domains in Dendrolimus kikuchii.pdf]

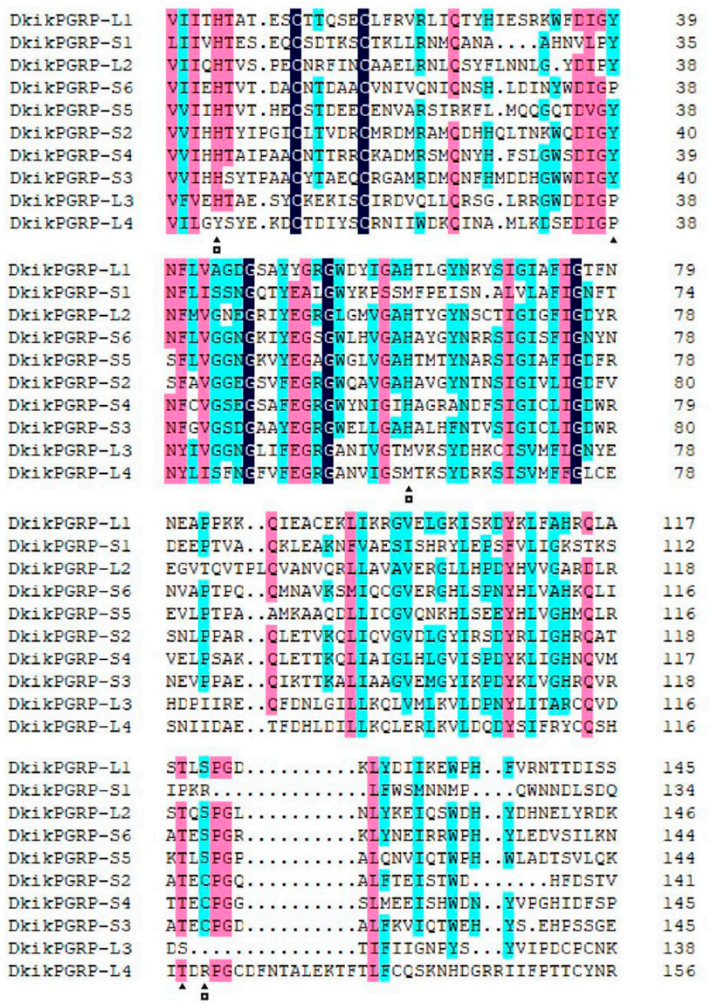

Figure S1. Multiple sequence alignment of PGRP domains in *D. kikuchii*. Black shading indicates identical amino acids; blue and pink shading indicates similar residues. Triangles (△) mark amidase catalytic sites, and squares (□) mark Zn^{2+}-binding sites.
